# Mr. Thomas's Observations on Dr. Cuming's Paper

**Published:** 1806-01

**Authors:** Hugh Thomas

**Affiliations:** Exeter


### *To the Editors of the Medical and Physical Journal.*

GENTLEMEN,

I Usually receive great pleasure in the perusal of your Journal, as a repository of curious Cases, and as conveying much information in the different branches of the profession; though, perhaps, if you were sometimes to exercise your power of selection to a larger extent, it might be no great disappointment to your readers. The recording of common observations and trivial cases, can excite very little interest in the public mind; but if the Authors think proper to deduce from them conclusions which appear to originate from an error in judgment, and endeavour to establish their inferences and opinions as an authority thority in practice, they become fair objects of criticism. On this ground I shall request your insertion of the following remarks on Dr. Cuming's communication, inserted in your Number for November.

Though I hope, in common with the Doctor and the greatest part of your readers, I may have learnt "how to appreciate local bleeding in topical inflammation," yet I must, contrary to his fond expectation, take the liberty to assure him, that I have really considered "the time employed in reading his paper as mispent, nearly one whole page of it being occupied with conveying to your Readers the wondrous information of the *partiality of leeches* for blood, and that it will prove an excellent bait to apply some on the skin, where it is intended they should attach; for the learned Doctor gravely acquaints us, that when the animal "tastes the blood, it generally fixes on the spot immediately, provided *we are not such fools* as to mistake the tail for the head;" but in this respect we have happily the comfort of the Doctor's opinion, that there is "no *vast* difficulty in discovering the one from the other." But to prevent even the possibility of disappointment, we are also informed of another "nouvelle" part of the process, that blood may readily be obtained for this purpose by, (wonderful to relate,) "tying a piece of thread or tape round the end of one's finger, and plunging the point of a needle into it"! And for all this knowledge, the Doctor modestly acquaints us, "he is indebted to a man in Romsey." Seriously, Mr. Editors, if all this had been "treasured up, miser-like, in the rusty coffers of the Doctor's own soul, I do not think the improvement of your Readers would have suffered any very considerable diminution.

I will now take the liberty of proceeding to his case of Ischuria; the recital of it makes one shudder at his patient's narrow escape from imminent danger, but in which I cannot see that the Doctor has much reason to plume himself on his knowledge and dexterity.

Mr. C. the subject of this case, it is said, "had not passed any urine for many *hours*;" from which I think we may infer, that Dr. Cuming's assistance was requested as soon as the pain and irritation, in consequence of the retention, became very severe. On the evening of the *same* day, the introduction of the catheter was first attempted, when, he tells us, "he was foiled in his attempt, after trying every possible method that was likely to render his endeavours efficient, and that the catheter passed with great ease, *apparently*, as far as the membranous part of the ure-

 thra thra or to the prostrate gland." Now comes the mischief, for we are next informed, that "by using a steady, firm, and cautious force, he felt the sensation of something giving way to the point of the instrument, impressing him with the idea that bands were formed across the passage, from the exudation of coagulable lymph, or that the passage was nearly or totally obliterated by surrounding inflammation." The instrument is then said to have "*advanced suddenly for the space of half an inch, and on withdrawing the stillette, nothing but a few drops of blood followed.*"

From the foregoing statement, does it not amount to more than a presumption, that the catheter was pushed through the membranous part of the urethra, between the bladder and rectum, or into the prostrate gland. The time elapsed since the commencement of the disorder is only described as being many hours, it can therefore be scarcely thought sufficient for the exudation of "coagulable lymph so as nearly or totally to obliterate the passage," or for the "formation of bands across it," so very firm as to resist the force with which they were assailed; and which, though the Doctor applies to it the epithet *cautious*, he observes, "*it required more than ordinary fortitude for his patient to bear.*"

The reader cannot be surprised that he "now deemed it prudent to desist."

After bleeding and administering an opiate, he directed his patient to be put into the warm bath, which (fortunately for the world) suggests to the mind of Dr. Cuming another GREAT DISCOVERY, equal in importance to the man of Romsey's with his leeches, that an *oval tub* is far more convenient for bathing a person in a horizontal position than a round one! This plan, however, not proving successful, he wisely determines on the following day to have recourse to the catheter, but for the purpose, it is said, of "facilitating its passage; though it would seem his patient could not be placed in the most convenient posture in the world, or the most favourable to the Doctor's *exertions*," he resolves to take the *advantage* of introducing it, whilst his patient remained in his favourite *oval tub*; but even in this *advantageous* posture, he "met with resistance so considerable as to stagger his fortitude;" however, under the "blessing of Providence, his endeavours were at last crowned with success: not, I imagine, from having forceably broken down any "bands formed across the urethra," but from having, after repeated trials, fortunately directed the instrument into the natural passage.

In

In confirmation of the opinion I have formed from the statement of this case, we find that the patient now complained of (what cannot be wondered at) a *strange sensation in the urethra*, and a pain and numbness in the course of the crural nerve, which, however it be accounted for, he dated from the *first attempt* which Dr. Cuming made to draw his water off, and it was succeeded on the following evening, as might be expected from an injury done to the urethra at that part, by a sense of heat and fullness about the neck of the bladder, not a word of which had been mentioned before during the formation of those bands across the urethra, or the exudation of the coagulable lymph, so as "*nearly or totally to obliterate the passage*." Nor does it appear that the *idea* of inflammation had made, before this time, any *very strong* impression on his mind, as it was now only that the leeches were first applied to the perineum. "The relief, however, obtained by them was so evident, as to enable the Doctor to announce another GREAT DISCOVERY to the world, "which, he says, neither teachers of medicine, nor authors, have inculcated," that the application of leeches to the neighbourhood of an inflamed part is "of utility!"

I shall now endeavour, which is the great object in my soliciting the publication of this paper, to counteract the impression which Dr. Cuming's remarks on the introduction of the catheter, have a tendency to make on the inexperienced practitioner, by contrasting them with the excellent observations of Mr. Hey in his Practical Observations in Surgery.

Though the force in passing the instrument is mentioned by Dr. Cuming to have been firm, cautious and steady, yet taking into consideration the *general* description and his observations on the whole case, it evidently conveys the impression of a greater exertion being required than can be admitted to be prudent or necessary, and which exertion, I think, I have sufficiently proved to have been the source of the chief difficulty in the treatment of this case, from which he deduces his opinion. He endeavours to fortify the mind of the practitioner, and prepares him to expect the operation to be a most painful one, requiring at times more than ordinary fortitude for his patient to bear; and by following the Doctor's example, he is to persevere even when he perceives the sensation of something giving way to the point of the instrument. Dr. Cuming concludes his paper by acquainting the reader, "that he is inclined to suppose that many cases have terminated fatally from want of *perseverance* and *fortitude* on the part

 of of the surgeon," and advises him to be "*deaf to the intreaties of the patient,* when he desires him to desist from pursuing those means which alone can insure success." "Many opportunities of shewing his conciliatory kindness and attention will occur, without risking the life of his patient by cowardly concessions."

How opposite is all this recommendation of force, perseverance, and fortitude, to the language of Mr. Hey, who, after describing, perhaps in the best possible manner, the method of performing this operation, and the cause of the laceration of the urethra, proceeds (page 388.) "Whatever method of performing this operation is pursued, the catheter should be introduced with the *greatest gentleness.* When any obstruction occurs, the design of the surgeon should be to *evade* rather than *overcome* it. Unsuccessful attempts may render a case *extremely difficult,* which was *not so before.* I wish to impress upon the mind of my reader that a *moderate* force, improperly directed, is capable of injuring the urethra in such a manner as to render the operation almost (and without a just knowledge of the injury altogether) impracticable. It must be obvious to every surgeon, that *long continued* or *violent attempts* have a tendency to increase the inflammation of the urethra. But the accidents to which I mean particularly to direct the attention, are the formation of a kind of pouch in the urethra, and the *laceration of its membranous part.*" Which latter accident, he afterwards adds, "he is persuaded occurs more frequently than is commonly imagined, and at times when no great force is used." The reader will, however, find the subject amply elucidated in the work itself, which contains more original information on this and a variety of other subjects in surgery, than any other book with which I am acquainted.

I am, &c.

*Exeter, Nov. 24, 1805.* HUGH THOMAS.

